# Pink salmon distribution in Sweden: The calm before the storm?

**DOI:** 10.1002/ece3.9194

**Published:** 2022-08-15

**Authors:** Thomas A. B. Staveley, Ida Ahlbeck Bergendahl

**Affiliations:** ^1^ Department of Aquatic Resources, Institute of Freshwater Research Swedish University of Agricultural Sciences Drottningholm Sweden

**Keywords:** invasive species, North Atlantic, river, salmonid, temperate

## Abstract

Pink salmon distribution has recently expanded substantially across northern Europe. On the Swedish west coast, relatively few pink salmon have been observed to date; nonetheless, a notable rise in 2021 (70 observations). However, with no national monitoring together with a ceased Atlantic salmon commercial fishery, there is little opportunity to understand the extent of the spread in this region. Here, we present the current data and address the need for future monitoring and research in order to understand the potential impacts of this invasive species in Sweden's aquatic ecosystems.

## Graphical abstract

Pink salmon distribution has recently expanded substantially across northern Europe. In Sweden, however, there is no national monitoring and together with a ceased Atlantic salmon commercial fishery, there is little opportunity to understand the extent of the distribution. Here, we present the current data and address the need for future monitoring and research in order to understand the potential impacts of this invasive species in Sweden's aquatic ecosystems.

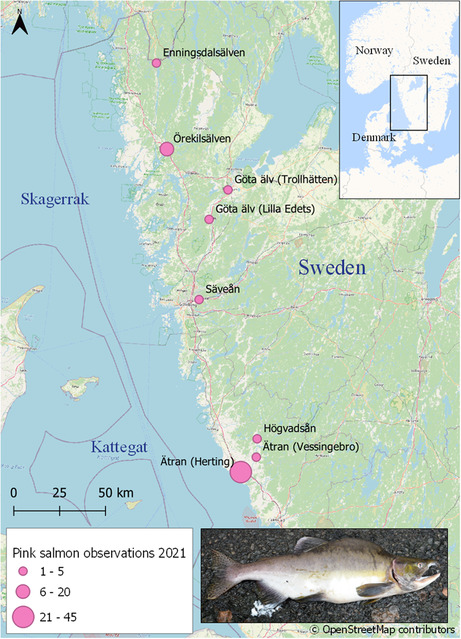

## 1 INTRODUCTION

Pink salmon (*Oncorhynchus gorbuscha*) have been observed in Swedish waters in relatively low numbers throughout recent years. However, the current increase across some countries in northern Europe, particularly in 2017, called for closer investigations into the spread, and potential impacts, of this fish which is native to the Pacific Ocean (Armstrong et al., 2018; Millane et al., 2019; Mo et al., 2018; Nielsen et al., 2020). The Pink salmon was introduced in 1956 to the Kola Peninsula in Russia and was subsequently stocked until 1998 (Gordeeva & Salmenkova, 2011). It has now spread in unprecedented numbers, in particular to northern Norway (see Sandlund et al., 2019). In 2021, catch data from Norway reported over 207,000 pink salmon in coastal and freshwater catches (Statistics Norway). Most fish were caught in the Troms and Finnmark County Municipality (most northern part of Norway) through targeted trap and net fishing. This is indeed an enormous increase from the ca. 10,000 individuals that were reported in Norway from 2017 (Mo et al., 2018).

In Sweden during 2021, pink salmon observations were their highest to date; a total of 70 individuals were reported from six rivers across four drainage basins. Even though these numbers are a fraction of those compared to Norway, this is nonetheless expected to be a considerable underestimate since there is no commercial salmon fishery on the Swedish west coast thus most fish go unnoticed. They may, however, be caught in by‐catch in fisheries targeting other species, though no pink salmon has been reported during the last 4 years (Data: Swedish Agency for Marine and Water Management). Pink salmon are now known to reproduce in some European rivers in, for example, Norway and the United Kingdom (Armstrong et al., 2018; Mo et al., 2018), but there is no reported evidence of successful reproduction yet in Swedish waters. The population of pink salmon occurring in northern Europe has mainly an odd year spawning cycle where adult spawners ascend European rivers every other year, for example, 2019, 2021, where they die upstream after spawning. Therefore, the next migration of pink salmon is expected in 2023.

This paper reports pink salmon observation data from Sweden between 2017 and 2021, while also addressing gaps in data and knowledge. In addition, we propose some initial research ideas to gain a better understanding of the distribution and reproductive potential of the pink salmon in Swedish waters.

## 2 METHODS

There is currently no national monitoring of pink salmon in Sweden; therefore, we can only rely on observations and reports from the public (e.g., recreational fishers), local authorities, and the few monitoring cameras already in place in some rivers. Current advice from the Swedish Agency for Marine and Water Management states that all caught pink salmon must be killed immediately and not put back into the water. All findings and observations should be reported to the local County Administrative Board (website: lansstyrelsen.se) and the Swedish Species Information Center (website: rappen.nu) at the Swedish University of Agricultural Sciences. However, as this reporting system is not always well known to those willing to report a finding, data have been reported to various authorities and organizations with varying degrees of information and quality; thus, data presented here are not always complete. Data presented here were collected from Fiskevårdsteknik AB, Swedish Species Information Center, Swedish Anglers Association (Sportfiskarna), County Administrative Boards of Västra Götaland, Halland and Skåne and Sven‐Erik Möller consultancy firm. Note, some of these data are from the public via the above organizations.

## 3 RESULTS

**FIGURE 1:**
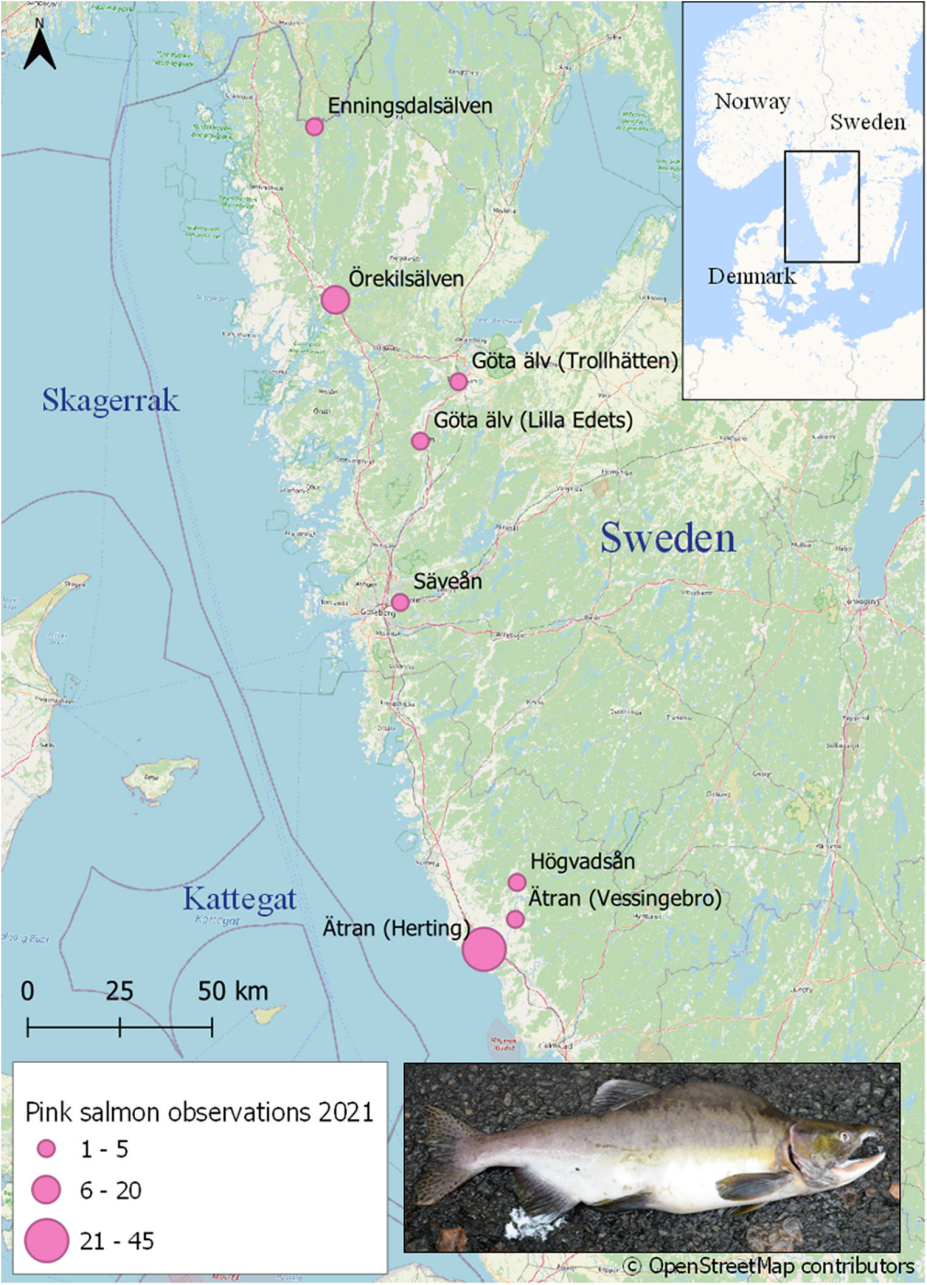
Pink salmon (*Oncorhynchus gorbuscha*) observations from the Swedish west coast during 2021. The size of the circle relates to the amount of reported observations. For the river Enningdalsälven, river Örekilsälven, and river Säveån, the exact point of observation is not known. Inset: A mature male pink salmon caught at Högvadsån in 2017 (photo: Hans Schibli).

Of the 70 observations and reported findings of pink salmon in rivers on the west coast of Sweden in 2021 (Figure 1), 45 came from the camera system at Herting (fiskedata.se) in the river Ätran, 4.3 km upstream of the river mouth. The majority of these fish passed through the camera in July (*n* = 37) with few passing in June (*n* = 4) and August (*n* = 4). The average length of the fish were 50 cm (SD ± 6.7 cm) with the minimum and maximum being 36 cm and 64 cm, respectively (Data: Fiskevårdsteknik AB). Further upstream in the river Ätran at Vessingebro (19 km from the river mouth) a single male, 55 cm, 1.5 kg, was caught in July. Two individuals were also caught at Nydala, river Högvadsån (32 km from the river mouth), which is also the designated river monitoring site for the Atlantic salmon (*Salmo salar*) for Sweden's west coast populations within the European Commission's data collection framework. In the river Göta älv, three pink salmon were caught at Lilla Edet (56 km from the river mouth) (mean length 46.3 cm; mean weight 0.9 kg) and a further four upstream in Trollhätten (77 km from the river mouth) during August (Figure 1). These reports in Trollhätten are of particular interest as it shows these individuals must have first passed successfully through the fish passage at the hydropower plant at Lilla Edet before being caught further upstream. In the river Säveån, a tributary to the river Göta älv, four pink salmon were observed in July. In the northern part of the Swedish west coast, 10 observations (no additional data) were reported in the river Örekilsälven and one (length 50 cm) in Enningdalsälven, close to the Norwegian border, in July (Figure 1).

In 2019, there were a total of only five observations, all from the camera system in the river Ätran. Three were observed in July and two in August with an average length of 58.4 cm (SD ± 16.1 cm; Data: Fiskevårdsteknik AB). The second highest number of reported findings of pink salmon in Sweden occurred in 2017, totaling 46 fish. At the camera system in the river Ätran, 18 individuals were reported from June to July (average length 55.2 cm; SD ± 7.9 cm; Data: Fiskevårdsteknik AB). At Vessingebro, one pink salmon was reported, which is likely the first known report of this species on the west coast of Sweden (Swedish Species Information Center). One report of a male pink salmon was also found at Nydala, river Högvadsån. One dead female, that was suspected to have already spawned, was found in Fylleån (close to Halmstad) in September. This is probably the southernmost record of pink salmon on the west coast in recent history. In the river Göta älv at Lilla Edet, 20 pink salmon were reported to have been caught during July and August. Two were reported from the river Örekilsälven at Kvistrum (3 km from the river mouth) measuring 53 and 48 cm in length. The only pink salmon report that occurred outside of any river system was a 50 cm fish in July, at the coastal site of Ljungskileviken, near Uddevalla. There were no reports of pink salmon in 2018 and 2020 on the Swedish west coast. Finally, no observations have been reported in recent years from Skåne County, which forms Sweden's most southerly coast and borders access into the Baltic Sea. This suggests that pink salmon have yet to spread this far south, or that no sightings or reporting has occurred.

## 4 DISCUSSION

To date, we do not know with certainty the extent of pink salmon occurrence in Sweden, and it is challenging to assess potential impacts due to lack of data. Currently, we are reliant on observations mainly from the public (e.g., anglers), as well as from a few monitoring cameras and stations. Similarly, data on how far up the river systems pink salmon migrate are indicative. At this stage, an important question raised is: *does the presence of the pink salmon cause any threat to the native salmonid species*? Thus far, it seems largely undetermined in the literature from northern Europe the extent and severity of any posed threats. We highlight some impacts here as much is already thoroughly discussed in the literature (e.g., Petersson et al., 2018; Sandlund et al., 2019). From the known observations of pink salmon (June to August) in Sweden, it is obvious that they occur in the river systems at the same time as Atlantic salmon ascend for spawning (mainly May–October) and may therefore interfere with the spawning of the Atlantic salmon and also sea trout (*Salmo trutta*). However, there are few data regarding the spawning success of pink salmon and possible interactions with salmonid species in northern Europe so far (Armstrong et al., 2018; Sandlund et al., 2019). Additionally, disease associated with pink salmon is also largely unknown thus further investigations would be needed in the future. Nonetheless, pink salmon eggs (Dunlop, Eloranta, et al., 2021) and fry (Mo et al., 2018) may serve an important nutrient source for young native salmonid populations while in the river environment. Recent research in Norway also postulates that dead post‐spawning adults can serve as additional nutrient sources in the aquatic and surrounding terrestrial ecosystems (Dunlop, Wipfli, et al., 2021), thus leading to increased nutrient availability.

Finally, we propose some initial ideas for monitoring and research in order to gain a thorough understanding of the distribution and impact of pink salmon in Sweden:

1. *Extent of spread*: The first step should be to conduct a comprehensive assessment of the geographic spread of pink salmon. This would determine which rivers they have migrated to and how far upstream they have ascended. Primarily including river systems on the Swedish west coast draining into the Atlantic Ocean but also systems heading into the entrance of the Baltic Sea, around the southern coast of Sweden. Suggested methods such as environmental DNA (Gargan et al., 2021) and electrofishing (depending on the accessibility of the river) would be advantageous. Increasing public awareness and willingness to report pink salmon would also be beneficial in documenting the distribution. Establishment of citizen science projects to highlight correct species identification and the need for data collection, particularly as dead fish after spawning may be more easily encountered by the general public.
2. *Reproductive success*: When the distribution knowledge of adult pink salmon is acquired, it is imperative to obtain information on the spawning and reproductive success in those specific rivers. However, together with the large variation in the timing of hatching (winter/early spring; Sandlund et al., 2019) and the relatively short time where the young smolt are thought to reside in freshwater before heading out to sea (compared to the Atlantic salmon where 82% of smolts migrate as 2 year olds in this region; Ahlbeck Bergendahl & Staveley, 2022), this poses substantial challenges in monitoring success. Indeed, this would require considerable resources, over space and time, to understand the extent of the spread and the reproductive success of the pink salmon in Sweden. Methods such as eDNA, electrofishing, nets, and smolt traps would be useful to detect pink salmon smolt in the river systems. Further research would also be needed to assess the survivability of the young pink salmon as they migrate downstream and into the coastal and marine environment.
3. *Increased connectivity*: As Sweden's hydropower facilities are currently undertaking a re‐trial assessment, migration barriers may be removed or measures implemented for increased connectivity in rivers (e.g., fish passages). This would allow easier movement for native migratory fish; however, also opening up the river upstream giving access to invasive species such as the pink salmon. Such potential effects of increased connectivity could be monitored (e.g., eDNA, tagging, cameras) before and after barrier removal, as well as understanding the spawning site selection by pink salmon, to understand movement potential up and downstream.

Only once we know the distribution and spawning success of the pink salmon in Sweden can we begin to uncover the potential impacts posed, thus leading to informed management action and mitigation measures. As others have mentioned (Jonsson & Jonsson, 2018), perhaps, there are little or no negative threats associated with the pink salmon, therefore suggesting that mitigation measures would not be needed at this time. However, whatever the outcome, the above suggestions should be considered in relation to the theory behind the “invasion‐curve” (e.g., Ahmed et al., 2022) where one can estimate the chances of eradication and mitigation in relation to time and spatial distribution of the introduced species.

Evidently, given our current lack of knowledge, together with recent explosions of pink salmon numbers in neighboring Norway, it would be anything other than cautious not to increase monitoring efforts of pink salmon in Sweden.

## AUTHOR CONTRIBUTIONS

**Thomas A. B. Staveley:** Conceptualization (equal); data curation (lead); investigation (equal); project administration (lead); visualization (lead); writing – original draft (lead). **Ida Ahlbeck Bergendahl:** Conceptualization (equal); data curation (supporting); investigation (equal); project administration (supporting); visualization (supporting); writing – original draft (supporting); writing – review and editing (equal).

## CONFLICT OF INTEREST

The authors declare no competing interests.

## Data Availability

Data available through Dryad data repository, doi: https://doi.org/10.5061/dryad.m37pvmd54.
